# A case report on delayed diagnosis of perforated Crohn’s disease with recurrent intra-psoas abscess requiring omental patch

**DOI:** 10.1016/j.ijscr.2019.11.021

**Published:** 2019-11-19

**Authors:** David Gao, Melissa G. Medina, Ehab Alameer, Jonathan Nitz, Steven Tsoraides

**Affiliations:** Department of Surgery, University of Illinois College of Medicine Peoria, 624 NE Glen Oak, Peoria, IL 61603, United States

**Keywords:** Perforating Crohn’s disease, Psoas abscess, Omental packing, Case report

## Abstract

**Introduction:**

Intra-abdominal abscesses associated with Crohn’s disease (CD) can rarely occur in the psoas muscle. An intra-psoas abscess is prone to misdiagnosis because its location mimics other diseases, like appendicitis and diverticulitis [1].

**Presentation of case:**

We present the case of a 25-year-old female with an 11-year history of CD, previously well-controlled on Remicade, who presented with right lower quadrant (RLQ) pain and CT findings of a right psoas abscess initially attributed to perforated appendicitis. Two percutaneous drainages pre-ileocecectomy, laparoscopic ileocecectomy, three percutaneous drainages post-ileocolectomy, and evidence of a recurrent abscess prompted diagnostic laparoscopy. The abscess was unroofed and debrided. A flap of omentum was used to fill the abscess cavity. A comprehensive literature search was performed using the terms ‘Crohn’s abscess’, ‘intra-psoas abscess’, and ‘omental patches’ in Medline and on PubMed.

**Discussion:**

We attribute the abscess’ recurrence to possible epithelialization of the abscess cavity. Intra-psoas abscesses, albeit rare, are a known manifestation of CD. Percutaneous drainage is the initial standard of care, although diagnosis can be difficult given its association with several diseases, which can delay definitive treatment. We summarize a recently proposed and agreed upon treatment scheme for the management of the Crohn’s patient with an abdominal abscess. We also propose the novel technique of omental packing in abscess management.

**Conclusion:**

Clinician awareness must be heightened for perforating CD in the setting of abscess refractory to either multiple drainage procedures, although care should be taken to individualize treatment to each CD patient who presents with an abdominal abscess.

## Highlights

• Psoas muscle abscess can be misdiagnosed for other etiologies, such as appendicitis.
• The current management of a Crohn’s abscess is debated.
• Treatment is influenced by abscess size, quantity of abscesses, and response to antibiotics and/or percutaneous drainage.
• Our patient, whose abscess was managed with the novel use of omental packing, has not demonstrated abscess recurrence.

## 1 Introduction

In patients with Crohn’s Disease (CD), two findings include intra-abdominal abscess (affecting 10–30% of patients with CD) and fistulas (affecting 17–50% of patients) [[2], [3], [4]]. A rare location for an abscess is on the psoas muscle, affecting between 0.4–4.3% of patients with CD [5]. Psoas abscesses are both rare and often complicated by misdiagnosis, due to their tendancy to mimic other disease processes [1]. Fistulas associated with CD most commonly present in the perianal region (54%), entero-enteric (24%), and recto-vaginal (9%) regions [3]. Reports of both psoas abscess and psoas-enteric fistula have only surfaced a handful of times [6]. We present a rare case of a 25-year old female with CD, psoas abscess, and psoas-enteric fistula, who was managed in an academic institution. She was initially misdiagnosed with acute appendicitis, thus delaying appropriate treatment. This work has been reported in line with the SCARE criteria [7].

## 2 Presentation of case

A 25-year old Caucasian female with a 10-year history of well-controlled CD, on infliximab, presented to a surgical outpatient facility with right lower quadrant (RLQ) abdominal pain. Her history since her diagnosis had been insignificant, and her symptoms prior to the appointment were well-managed with infliximab. She follows a vegan diet.

Two weeks prior to presentation at our facility, she walked into the emergency room with a two-week history of shortness of breath, fever, chills, abdominal pain, and diarrhea without hematochezia. Her history since her diagnosis had been insignificant. Septic workup included a CT scan which revealed a RLQ psoas abscess posterior to the cecum that had been attributed to perforated appendicitis. She then underwent CT-guided drainage of the abscess by the interventional radiology (IR) department. Cultures were positive for *S. intermedius*, and she was subsequently treated with amoxicillin-clavulanate.

Nine days after her first IR drain, the patient presented for re-evaluation due to persistent symptoms. Given her history of infectious abscess, infectious disease (ID) was consulted, and she was started on piperacillin/tazobactam and vancomycin. Her symptoms did not resolve, and a subsequent CT scan confirmed the abscess had localized to the psoas muscle, prompting another image-guided drainage.

Following this second drain, she continued experiencing low-grade fever, pain, and diarrhea now concerning for retrocecal fistula. 14 days post-admission, she underwent a laparoscopic ileocecectomy and drain removal and ileocecectomy with primary anastomosis. We discovered additional areas of perforation and multiple abscesses smaller than 3 cm (cm). Her percutaneous IR drain was removed during the procedure. Afterwards, her signs and symptoms of infection abated almost immediately, and she was discharged on oral metronidazole.

Two weeks later, the patient presented with a four-day history of RLQ pain and abdominal cramping. A flare-up of her known CD was suspected. The patient was continued on infliximab and monitored; however, three days later (one month after the initial encounter) she was admitted with acute elbow and knee pain. Her antibiotics were switched to daptomycin, ertapenem, and micafungin. Her pain improved, and she was discharged five days after admission. Two weeks after this visit, she continued care at a separate hospital, where they placed her third IR drain.

**Fig. 1:**
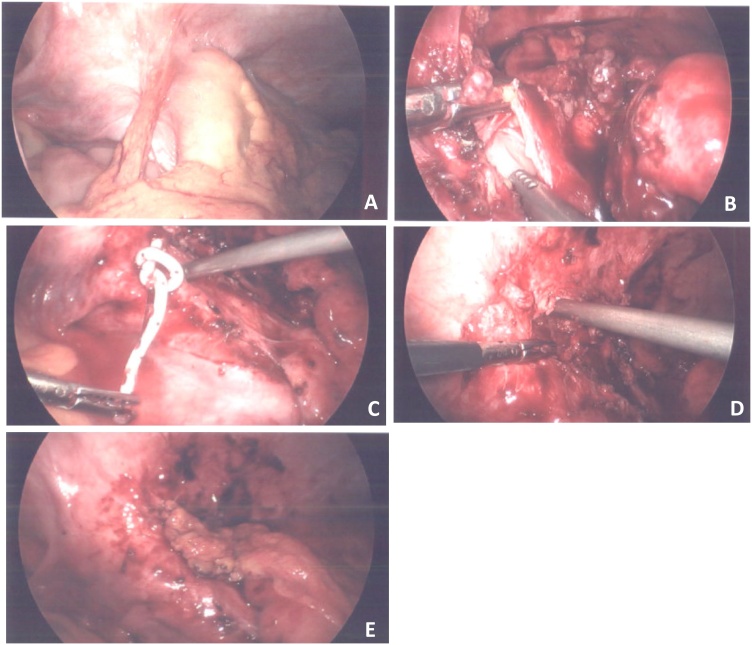
**(A)** Bowel and omental adhesions to abdominal wall **(B)** Abscess cavity localized by instilling sterile water in the existing IR-placed drain **(C)** Tip of IR drain (Pigtail) brought out after unroofing of the chronic abscess cavity **(D)** Completion of abscess cavity unroofing after IR drain was externally removed **(E)** Omental flap used to pack the unroofed chronic abscess cavity and secured in place with sutures.

One month later (two months after our initial encounter), she presented to the ED with fever, abdominal pain, and non-bloody diarrhea. Due to concern for a recurrent fistula, she underwent a diagnostic laparoscopy. Significant, dense adhesions were encountered in the right abdomen between the distal ileum and lateral abdominal wall (Fig. 1A). The anastomosis from the prior operation was found to be intact, and no obvious cause for the recurrent psoas abscess was seen. To access the abscess, the existing IR drain was used as a guide. The abscess was unroofed and its cavity thoroughly irrigated with sterile water (Fig. 1B–D). To prevent recurrence, a flap of omentum was freed, packed within the abscess to encourage healing, and secured to the cavity’s edges (Fig. 1E). No pus was noted in the abdomen. She recovered well, and four days following the procedure, she was discharged with a course of oral antibiotics. Two months later, her psoas abscess was imaged via ultrasound and confirmed to have healed with no drainable fluid component.

## 3 Discussion

Our patient was initially diagnosed with perforated appendicitis causing a psoas muscle abscess. Given the abdominal pain and abscess at initial presentation, as well as pain extending to the back, fever, and increased CRP, appendicitis was not inappropriate to include on the differential. Ruptured appendicitis was questioned, however, when the patient presented with these persistent symptoms. Moreover, whereas a perforated appendicitis might spread its contents throughout the abdominal cavity, our patient’s abscess localized to the psoas muscle.

Current management of a Crohn’s abscess is debated. No randomized control trial comparing PD, surgery, and antimicrobials has to date been conducted. A recent meta-analysis comparing PD and surgery found that PD was associated with a significantly higher likelihood of abscess recurrence (OR = 6.544) but found no difference in complication rates (OR = 0.657) or length of stay (difference in means = −1.006) [8]. Some studies have shown that initial PD makes follow-up surgery more successful [9]. Others have shown that initial surgical management to be more effective in reducing post-operative complications [10]. Other studies retrospectively examining an interventional group (drainage or surgery) and medicine-only antimicrobials group have found no significant difference in rates of abscess recurrence or nonresolution [10].

The lack of an agreed upon treatment course is further complicated by different recommendations. The *European Crohn’s and Colitis Organization* recommends that all spontaneous abscesses be managed via broad-spectrum antibiotics and imaging-guided PD [11]. Other physician groups recommend treatment be guided by more factors, such as abscess size, presence of a fistula, immunosuppression, and abscess persistence. For complicated CD, defined as the presence of a structure, fistula, post-op abscess, or a spontaneous abscess larger than 3 cm, these groups recommend antibiotics or PD, followed by surgery should the abscess persist [2]. The American College of Radiology recommends a similar approach: treating abscesses smaller than 4 cm with antibiotics and larger abscesses with PD, high-dose steroids, bowel rest, and hyperalimentation [12].

These conflicting reports found in our comprehensive literature search make a definitive conclusion about treatment difficult. A treatment algorithm recently proposed by *Carvalho et al.* and that agrees with current American College of Radiology guidelines takes into account patients’ abscesses characteristics, medication history, and disease progression [13]. They recommend initial imaging, best performed via ultrasonography or CT, to determine the size of the abscess [14]. Size is a good predictor for success in medication-only therapy, making imaging an excellent first step in determining a conservative, antibiotics approach versus procedural intervention [12]. Small (<=4 cm) abscesses can be managed by medical treatment using either extended spectrum beta lactamases (ESBL) or ciprofloxacin/metronidazole, both of which have shown being well-tolerated and efficacious [15]. Should complications arise during treatment (persistent abscess, stricture development, or fistula development), surgery is recommended.

For abscesses greater than 4 cm, PD is initially done to control peritoneal contamination [16]. If initial antibiotics and PD were successful, and an abscess is discovered during planned resection and primary anastomosis (as in our patient), the patient can still proceed with PD [17]. Should complications arise after PD, surgery is recommended [18]. In a patient for whom PD is not possible (with a comorbid interloop, intra-mesenteric, or multiloculated abscess), immediate surgery has demonstrated efficacy [19]. Patients successfully treated should then continue their routine immunomodulator or biological treatment (TNF-alpha inhibitor).

Omental packing is a technique we did not find to be discussed in any guideline for treating abdominal abscess treatment in a CD patient. We utilized the omentum to encourage wound healing following debridement of the abscess capsule. A 2012 study retrospectively examining 45 patients who had undergone abdominoperineal resection for lower rectal adenocarcinoma found that both wound infection in the packed wound group was significantly lower (5% compared to 32%) and duration of stay shorter (17.8 days compared to 21.0 days) [20]. To date, our patient remains symptom-free and continues on her biologic therapy for Crohn’s. Due to its efficacy in our patient, omental packing ought to be further scrutinized in the treatment of a CD patient with abdominal abscess.

## 4 Conclusion

Management of an abscess in CD should be guided by multiple factors that may influence treatment management. One novel technique we propose is the use of omental packing to prevent abscess recurrence. In our opinion, it may be of worth to investigate its use in addition to traditional management of an abdominal abscess.

## Sources of funding

This research did not receive any specific grant from funding agencies in the public, commercial, or not-for-profit sectors.

## Ethical approval

This study is exempt from ethical approval in our institution.

## Consent

The head of our medical team has taken responsibility that exhaustive attempts have been made to contact the family and that the paper has been sufficiently anonymised not to cause harm to the patient or their family. A signed document to this effect, has been uploaded.

## Author contribution

Steven Tsoraides, Corresponding Author, contributed to the paper as follows: conceptualization, investigation, resources, writing – review and editing, visualization, supervision, and project administration.

Melissa Medina contributed to the paper as follows: investigation, resources, writing – original draft, writing – review and editing, visualization, supervision, and project administration.

Ehab Alameer contributed to the paper as follows: investigation, resources, writing – review and editing, and visualization.

Jonathan Nitz contributed to the paper as follows: investigation, resources, writing – review and editing, and visualization.

David Gao contributed to the paper as follows: investigation, writing – original draft, writing – review and editing, visualization, and project administration.

## Registration of research studies

Not required.

## Guarantor

The Guarantor is Dr. Steven Tsoraides, MD.

## Provenance and peer review

Not commissioned, externally peer-reviewed.

## Declaration of Competing Interest

The authors declare that they have no personal, financial, or professional relationships with other people or organizations that could inappropriately influence or bias our work.
